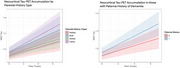# Parental history of memory impairment predicts tau‐PET accumulation in a preclinical population

**DOI:** 10.1002/alz70861_108687

**Published:** 2025-12-23

**Authors:** Mabel Seto, Hannah M Klinger, Gillian T Coughlan, Colin Birkenbihl, Hyun‐Sik Yang, Timothy J. Hohman, Elizabeth C. Mormino, Kathryn V Papp, Rebecca E. Amariglio, Dorene M. Rentz, Keith A. Johnson, Aaron P. Schultz, Reisa A. Sperling, Rachel F. Buckley

**Affiliations:** ^1^ Center for Alzheimer's Research and Treatment, Brigham and Women's Hospital, Harvard Medical School, Boston, MA USA; ^2^ Massachusetts General Hospital, Harvard Medical School, Boston, MA USA; ^3^ Brigham and Women's Hospital, Harvard Medical School, Boston, MA USA; ^4^ Vanderbilt Memory & Alzheimer’s Center, Vanderbilt University Medical Center, Nashville, TN USA; ^5^ Department of Neurology and Neurological Sciences, Stanford University, Stanford, CA USA; ^6^ Department of Radiology, Division of Molecular Imaging and Nuclear Medicine, Massachusetts General Hospital, Boston, MA USA; ^7^ The Athinoula A. Martinos Center for Biomedical Imaging, Department of Radiology, Massachusetts General Hospital, Boston, MA USA

## Abstract

**Background:**

Previous studies suggest that a maternal history of Alzheimer’s disease (AD) confers a greater risk for AD than paternal history through elevated Aβ‐PET, reduced brain glucose metabolism, and lower grey matter volume though these studies were primarily cross‐sectional, limiting causal inferences. Whether parental history of AD dementia impacts AD endophenotypes longitudinally is not fully described. We examine the effects of parental history of dementia on longitudinal measures of cognition and AD biomarkers in a preclinical population.

**Methods:**

Longitudinal Aβ‐PET (^18^F‐florbetapir), tau‐PET (^18^F‐flortaucipir), and cognition (PACC‐5) data from 1,693 cognitively unimpaired individuals from the Anti‐Amyloid Treatment in Asymptomatic Alzheimer’s (A4) clinical trial and the adjoining LEARN observational study were used for these studies (mean(SD)_age_=71.29(4.67), female=59%, *APOE*ε4+=35%; n=446 with tau‐PET). Parental history was self‐reported by participants and defined as “history of cognitive impairment and/or dementia.” Linear mixed‐effects models assessed the association between parental history (i.e., maternal/paternal) and the outcomes of interest covarying for the participant’s age, sex, and years of education where applicable. The following interaction terms were also examined: parental history*sex, parental history*APOEε4 allele count, and parental history*Aβ_continuous_ burden.

**Results:**

There was no effect of parental history on longitudinal Aβ accumulation or PACC‐5 scores. We found that participants with a paternal history of memory impairment or dementia accumulated tau faster than those without paternal history (β=0.01, SE=0.003, *p* =0.004, Figure 1). This association remained significant when covarying for *APOE*‐ε4 and *APOE*‐ε2 allele count. Tau accumulation was not affected by paternal age at symptom onset (*p* =0.175). We found no moderating effects. We observed no association between maternal history and tau accumulation.

**Conclusion:**

This is one of the first studies of parental history and longitudinal AD endophenotypes in a large sample of cognitively unimpaired individuals. Our findings that participant‐reported history of paternal history is associated with faster tau accumulation stands at odds with evidence suggesting greater risk of maternal history of dementia. However, a recent study by Ourry et al., also highlights a relationship between paternal history and tau. These findings could be driven by biological or survival bias differences in the sample and deserves further exploration.